# Mechanistic Insights into the Oxidized Low-Density Lipoprotein-Induced Atherosclerosis

**DOI:** 10.1155/2020/5245308

**Published:** 2020-09-15

**Authors:** Chainika Khatana, Neeraj K. Saini, Sasanka Chakrabarti, Vipin Saini, Anil Sharma, Reena V. Saini, Adesh K. Saini

**Affiliations:** ^1^Department of Biotechnology and Central Research Cell, MMEC, Maharishi Markandeshwar (Deemed to be University), Mullana, 133207 Ambala, Haryana, India; ^2^Faculty of Applied Sciences and Biotechnology, Shoolini University, Solan, 173212 Himachal Pradesh, India; ^3^Department of Microbiology and Immunology, Albert Einstein College of Medicine, Bronx, NY 10461, USA; ^4^Department of Biotechnology, Jawaharlal Nehru University, Delhi, India; ^5^Department of Biochemistry and Central Research Cell, MM Institute of Medical Sciences & Research Maharishi Markandeshwar Deemed to be University, Mullana, 133207 Haryana, India; ^6^Maharishi Markandeshwar University, Solan, 173212 Himachal Pradesh, India

## Abstract

Dyslipidaemia has a prominent role in the onset of notorious atherosclerosis, a disease of medium to large arteries. Atherosclerosis is the prime root of cardiovascular events contributing to the most considerable number of morbidity and mortality worldwide. Factors like cellular senescence, genetics, clonal haematopoiesis, sedentary lifestyle-induced obesity, or diabetes mellitus upsurge the tendency of atherosclerosis and are foremost pioneers to definitive transience. Accumulation of oxidized low-density lipoproteins (Ox-LDLs) in the tunica intima triggers the onset of this disease. In the later period of progression, the build-up plaques rupture ensuing thrombosis (completely blocking the blood flow), causing myocardial infarction, stroke, and heart attack, all of which are common atherosclerotic cardiovascular events today. The underlying mechanism is very well elucidated in literature but the therapeutic measures remains to be unleashed. Researchers tussle to demonstrate a clear understanding of treating mechanisms. A century of research suggests that lowering LDL, statin-mediated treatment, HDL, and lipid-profile management should be of prime interest to retard atherosclerosis-induced deaths. We shall brief the Ox-LDL-induced atherogenic mechanism and the treating measures in line to impede the development and progression of atherosclerosis.

## 1. Introduction

World Health Organisation (WHO) has enumerated quite a few diseases responsible for the disability and deaths in the industrialized world, among which cardiovascular diseases (CVDs) are the most common, invoking about 17.9 million deaths annually (WHO fact sheet). In-depth mechanistic knowledge is anticipated to lessen the effects and consequences of critical risk factors involved in this disease. Epidemiological studies imply that elevated level of low-density lipoprotein (LDL) (cholesterol carrier through the blood of 20-25 nm size) is the chief contributor to atherosclerosis [1, 2]. Atherosclerosis refers to the disease of arteries which commonly manifests as coronary heart disease (CHD) leading to myocardial infarction and cerebrovascular disease leading to stroke and other complications. Atherosclerosis is a complex, progressive, inflammatory disease that mainly occurs in subendothelial space (tunica intima) of medium to large-sized arteries at regions of disturbed blood flow or bifurcates [3–5]. The progression of atherosclerotic disease depends on the presence, degree, and persistence of risk factors like high-fat diet, smoking, hypertension, history of heart diseases, or diabetes [6–8] (Table 1). Experimental observations have explicitly pinpointed oxidized low-density lipoprotein (Ox-LDL), endothelium dysfunction, and oxidative stress as the most prominent risk factors in atherosclerosis [9–11].

Under normal physiological condition cell maintains redox homeostasis which plays a significant role in signalling and, any imbalance in this homeostasis may instigate a chain of reactions generating free radicals (reactive oxygen species termed here as ROS or reactive nitrosative species termed here as RNS) [11, 12]. Disturbed redox balance or the imbalance between reactive species and antioxidant system leads to the oxidative stress that damages biomolecules like proteins, nucleic acids, and lipids. Exposure to exogeneous chemical and physical agents (like environmental pollutants, radiation, UV exposure, or smoking) or endogenous enzymes (like NADPH oxidase, xanthine oxidase, cytochrome P450, or nitric oxide synthase (NOS) and metal ion catalysis (Fenton reaction)) generally results into increased ROS production [11, 13, 14]. Physical forces including oscillatory shear are also reported to have a share in vascular production of ROS [11, 15]. Lipids serve as primary targets of reactive species with important relevance to CVDs, and these lipids can be analyzed by several methods [13, 16, 17]. Lipid oxidation, commonly known as lipid peroxidation, is a process that involves peroxidation of phospholipids and cholesterol esters at the polyunsaturated fatty acid moieties (linoleate, arachidonate, etc.) by nonenzymatic mechanisms (ROS, RNS, or in presence of transition metals like Fe^2+^ and Cu^2+^) [18–20]. Maintenance of plasma LDL levels is important for CVD (23, 24). Additionally, the polyunsaturated fatty acids may be oxidized by lipooxygenase, cyclooxygenase, and cytochrome P450-dependent oxygenases producing ROS in the process. Lipid peroxidation progresses through 3 stages: initiation, propagation, and termination [21]. The free radical chain oxidation mechanism initiates with a lipid radical formation (abstraction of hydrogen atom from the CH_2_ group) leading to the propagation step where it reacts with oxygen generating lipid-peroxyl radicals that transform it into lipid hydroperoxides and finally, the reaction terminates once nonradical products are formed releasing oxygen [22]. It is quite difficult to quantify the products of lipid peroxidation because of the complexity of lipid peroxidation pathway and its by-products. New techniques like mass spectroscopy tools are now being used to at least meet some challenges [17].

**Figure 1:**
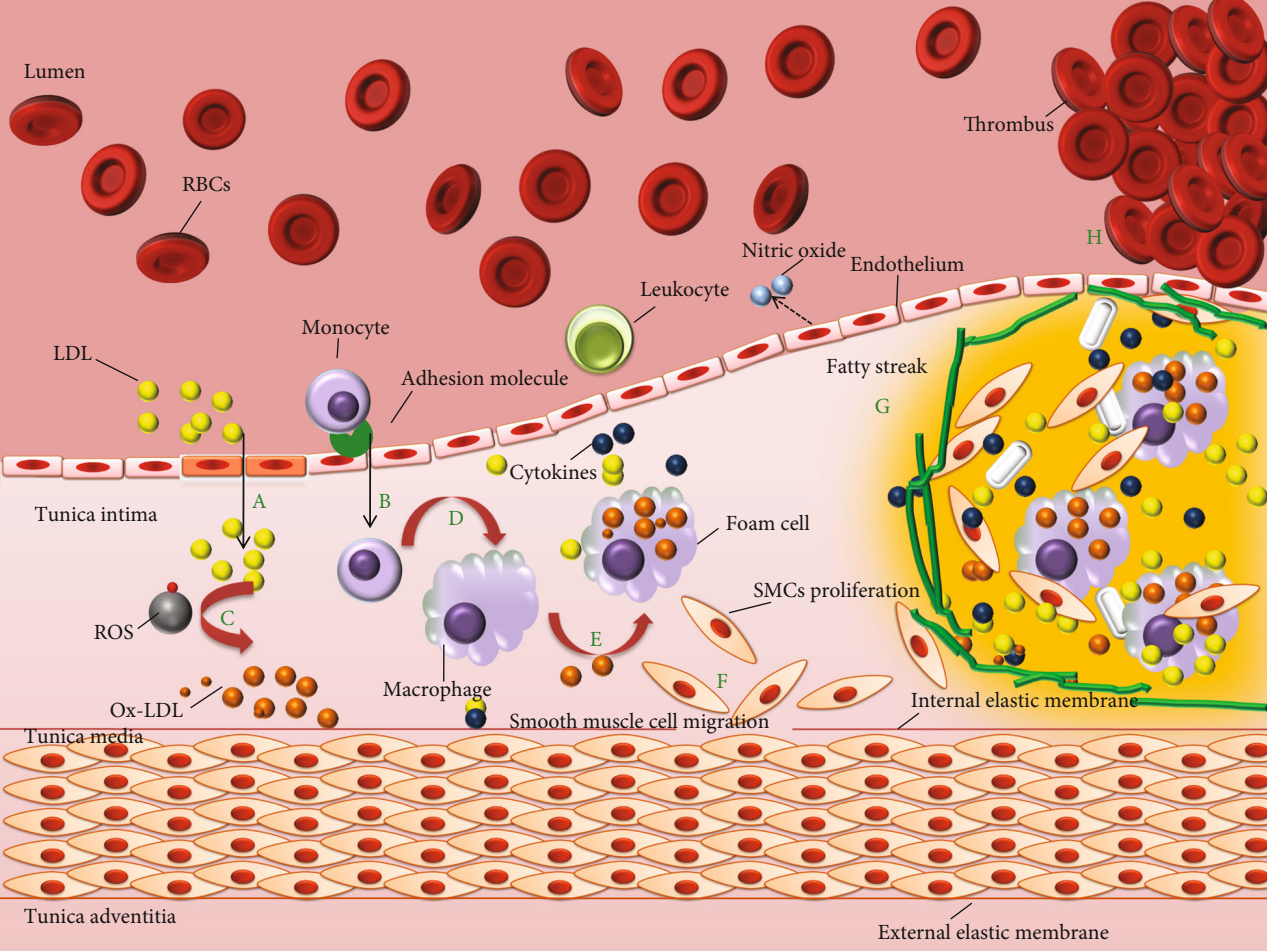
Schematic network of atherosclerosis process. The LDL in the blood stream passes through the damaged endothelium (caused by hypertension, high cholesterol, smoking, and hyperglycemia) gaining entry into tunica intima. Damaged endothelial cells being compromised express adhesion molecules that capture the monocytes. Monocytes enter into the intima producing free radicals which oxidizes the LDL. Oxidized LDL attracts more white blood cells (monocytes) and more immune cells to the site, macrophages engulf Ox-LDL particles becoming over laden and turning into foam cells. Foam cells die releasing its content outside that again is engulfed by other macrophages eventually building a large lesion area. Progression into this, lesion turns into plaque gradually accumulating calcium slats, smooth muscle cells (from tunica media), collagen, and the foam cells. The plaque is stable under the endothelium until the endothelium just above gets compromised. The damaged endothelium could no longer produce inhibitors for blood clotting making it more vulnerable to enter into the vessel lumen. The clot attached to the vessel wall would make a thrombus that may break causing stroke or myocardial infarction.

Under normal conditions, the plasma LDL is composed of triglycerides and cholesterol esters with an outer layer composed of phospholipids, free cholesterol, and apolipoprotein B (ApoB) that carry hydrophobic cholesterol through the blood [15]. Increase in the plasma LDL levels is generally associated with atherosclerosis [23]. Under oxidative stress, the oxidation of LDL occurs by the process of lipid peroxidation primarily involving the phospholipid molecules. In pathological conditions, ApoB-containing lipoproteins in the plasma penetrate through the damaged endothelium into vascular subendothelial intima getting oxidized by ROS [24, 25]. Under these conditions, the LDL is modified into Ox-LDL [26]. LDL retention in subendothelium confers monocyte recruitment to tunica intima, where they differentiate into macrophages. Ox-LDL serves as strong ligands for macrophage scavenger receptors (CD36, SR-AI/II, and SR-BI) that facilitate their entry into macrophages [27]. It is suggested that the oxidation of specific epitopes (OSEs) occur on Ox-LDL that are recognized by the cellular and humoral innate immune system and thus enhancing the internalization of Ox-LDL into macrophages [28]. Macrophages engulf Ox-LDL through its scavenger receptors and the accumulation of Ox-LDL into macrophages gives it a morphologic appearance of soap bubbles termed as foam cells which later result in atherosclerotic lesions leading to atherosclerotic plaque build-up, limiting the blood flow to the heart muscle (see Figure 1 for schematic diagram) [29, 30]. Very recently, a study was published providing insights into myeloid tribbles 1 that induces early atherosclerosis via extensive foam cell formation [31]. A native LDL cannot exert atherogenic mechanisms in vitro which implicate that to be pathogenic it must have been modified, explaining oxidative damage as a pro [32].

On the brighter side, high-density lipoproteins (HDLs), endogenous apolipoprotein E (ApoE), and ApoA-I proteins promote efflux of surplus cholesterol from the macrophage via specific transporters of the ATP binding cassette (ABC) gene family [33]. This efflux is the primary line of defence to protect against the progression of macrophage to foam cells. Further, antioxidants scavenge ROS combating atherosclerosis in the initiation period [34]. Efforts have been made to comprehend the mechanism of lipid peroxidation in developing effective therapeutic drugs and prevent its deleterious effects. Herein, we have discussed the role of distinct factors critical in the progression of atherosclerosis.

## 2. Oxidized Low-Density Lipoprotein as the Major Risk Factor in Atherosclerosis

**Figure 2:**
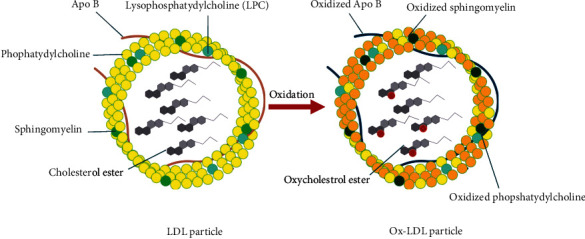
LDL and Ox-LDL Structure. The hydrophobic core of LDL is made of around 170 triglycerides, 1500 cholesterol esters, a hydrophilic coat composed of 700 molecules of phospholipids, about 500 molecules of unesterified cholesterol, and a single large copy of the apolipoprotein B (ApoB) of 500 kDa. Gaining entrance into the endothelium, LDL gets oxidatively modified by ROS.

Low-density lipoprotein is a chief carrier of cholesterol to the cells. High dietary fat adds to its abundance causing pathological complications above a threshold level [35]. It belongs to a heterogeneous group of particles having a mass of about 3000 kDa with a diameter of 220 nm [36]. The hydrophobic core of LDL is made of around 170 triglycerides, 1500 cholesterol esters, a hydrophilic coat composed of 700 molecules of phospholipids, about 500 molecules of unesterified cholesterol, and a single large copy of the ApoB of 500 kDa [37]. Structure of LDL and Ox-LDL is shown in Figure 2.

During oxidative stress, the LDL is modified to Ox-LDL [38]. In addition to this, lipoxygenase and phopholipase A2 enzymes alter LDL in a manner of getting easily recognized and engulfed by macrophages [39]. Speaking specifically in terms of atherosclerosis, ROS induces lipid oxidation, instigating the disease. LDL oxidation brings about certain modifications to the LDL structure giving it a higher density, hydrolysing phosphatidylcholine, amending lysine residues of ApoB, and degrading ApoB [37]. Polyunsaturated fatty acids (PUFAs) in the LDL are more prone to oxidation as compared to monounsaturated fatty acids (MUFAs) [40]. PUFAs encountering oxidation (enzymatic and nonenzymatic) convert into hydroperoxides further breaking down to generate more reactive aldehyde products and metabolites such as malondialdehyde and 4-hydroxynoneal which build adduct with Schiff-base lysine residue of ApoB, phosphatidylserine, and phosphatidylethanolamine [41]. ApoB in this context determines the generation and propagation of LDL oxidation. Additionally, the elevated temperature used while preparing meals increases the oxidation of cholesterol [42]. Nonetheless, oxysterols coming from the dietary sources into the bloodstream may implicate a fundamental role in the progression of this disease. Thereby, one cannot conclude the root embarking such progression and advanced studies need to be done.

Discoveries by Virchow and Windaus in the nineteenth century bring forth the crucial role of lipoproteins in atherosclerosis. Windaus discovered high cholesterol in human artery which thereafter confirmed by Nikolaj Anitschkow who produced atherosclerosis in cholesterol fed rabbits implicating dietary cholesterol as an important factor [43, 44]. Carl Muller revealed a compelling connection between heart attacks and plasma cholesterol giving the cholesterol research a new turn introducing the hypercholesterolemia [45]. Widespread theories put forward LDL penetrating the dysfunctional endothelium and retaining in the subendothelium lining as the hallmark of atherosclerosis [46]. The higher the LDL the faster the plaque evolves. Surprisingly, native LDL is not atherogenic and needs to be oxidatively modified, particularly ApoB, to instigate the disease while it stays in the subendothelium bound to glycosaminoglycans [47, 48]. Macrophages with their surface receptors recognize the modified LDL and engulf them to become lipid laden foam cells which secrete cytokines making the site more vulnerable to inflammation and instigating smooth muscle cell (SMC) proliferation [49–51]. Today, many publications could be found on Ox-LDL to provide evidence for its role in atherosclerosis which makes it a relevant candidate and somewhat apparent therapeutic target. Though very few studies could robustly prove the definitive decrease of LDL with dietary antioxidant supplements, it is believed that a combination of antioxidants, therapeutics, and lipid management could work wonder (discussed later in this review).

### 2.1. Stages of Ox-LDL-Induced Atherosclerosis Mechanism: Initiation to Thrombosis

**Figure 3:**
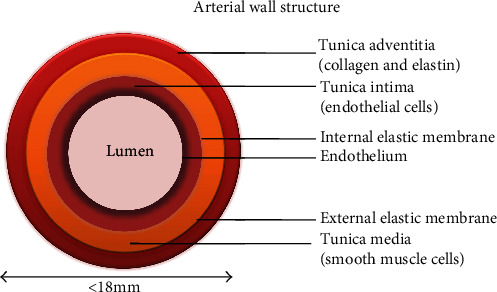
Artery is made of three layers: tunica intima, media, and adventitia.

**Figure 4:**
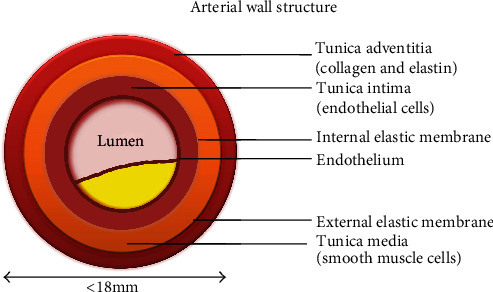
Atherosclerotic artery. Atherosclerotic artery has a plaque build-up in the subendothelium making the lumen too small for a smooth blood flow.

**Figure 5:**
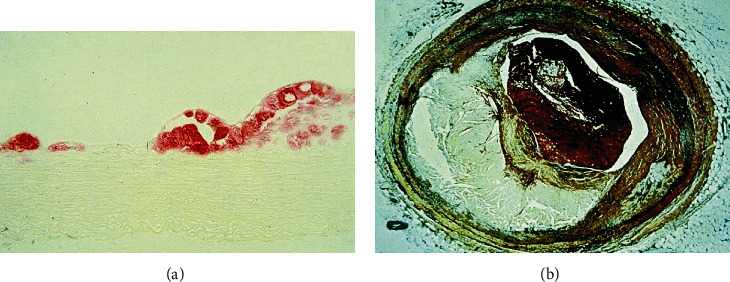
Early and late atherosclerotic lesions. The figure is reproduced from Glass and Witztum [56] (under the Creative Commons Attribution License/public domain) “(Reprinted (Atherosclerosis: The Road Ahead) with permission from Elsevier (License number 4825741069515)).”(a) Cross section of a fatty streak lesion from the aorta of a cholesterol-fed rabbit immune stained for a macrophage-specific marker (micrograph courtesy of Wulf Palinski). (b) Cross section through a human coronary artery at the level of a thrombotic atherosclerotic lesion causing fatal myocardial infarction.

Atherosclerosis originates with the subendothelial retention of ApoB 100, containing lipoproteins. LDL penetration through dysfunctional endothelium brings many factors into the picture (summarized from the initiation to calcification in this review). Broadly, the process can be classified into three stages: initiation, progression, and thrombosis. A human artery is composed of three layers: the tunica intima, the tunica media, and the tunica adventitia, as shown in Figure 3. Intima is lined with a single layer of endothelial cells called the endothelium and subendothelial extracellular matrix (consisted of collagen and elastin). Endothelium aids in regulating vascular tone, coagulation, and maintaining vascular homeostasis via highly regulated mechanisms with the function of nitric oxide, prostacyclin, and endothelin-1 [52, 53]. Numerous smooth muscle cells (SMCs) are found in tunica media which are organised concentrically within an elastin-rich cellular matrix to store the required kinetic energy for transmission of pulsatile flow [54]. The adventitia contains mast cells, fibroblasts, and a matrix containing proteoglycans and collagen. Internal and external elastic lamina separates the intima, media, and adventitia, respectively [55]. Nevertheless, the artery gets compromised with the interplay of Ox-LDL and other risk factors making hard and narrowing the lumen for a disturbed blood flow. An atherosclerotic artery with plaque is shown in Figure 4 and a cross section of early and late atherosclerotic lesion is shown in Figure 5 [56].

**Figure 6:**
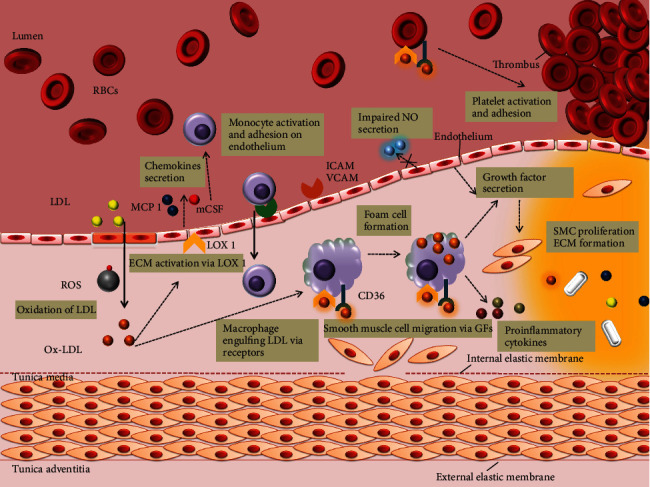
Schematic illustration of the role of Ox-LDL in atherosclerosis progression. Ox-LDL elicits atherosclerotic events right from their production in the subendothelium. Due to downregulated LDL receptors, the native LDL cannot be internalized by macrophages. Ox-LDL, via LOX 1 and other factors, activates endothelium for a number of events: adherence of LDL, monocytes, and platelets; secretion of chemokines and growth factors; production of ROS; impairing NO secretion; and so on. SRs, CD36, and LOX 1 help in the uptake of OX-LDL by monocyte-derived macrophages in the subendothelium. Growth factors mediate SMC proliferation and extracellular matrix formation. Platelet adherence and accumulation is also, in part elicited by Ox-LDL which results into a rupture prone thrombus.

Ox-LDL instigates atherosclerotic events throughout the disease progression, starting from endothelium dysfunction, white blood cell activation, foam cell formation, SMC migration and proliferation to platelet adhesion and aggregation (refer Figure 6).

### 2.2. Physiology of Endothelial Dysfunction: Initiation Stage

In normal body parameters, a healthy endothelium remains the primary regulator of vascular homeostasis [57–59]. It assists in keeping a balance between vasodilation and vasoconstriction, thrombogenesis and fibrinolysis, and inhibition and stimulation of SMC proliferation [60]. Nonetheless, it acts as a barrier between the circulating blood (in lumen) and the artery lining (endothelium) [61]. An intact endothelium experiencing laminar shear stress elicits signalling pathways to maintain glycocalyx layer, proliferation, and endothelial cell coaxial alignment [10, 62]. Nitric oxide synthase (NOS) is expressed via MEK5 signalling which promotes the nitric oxide (NO) production and further helping in the endothelium survival. The antiatherogenic role of NO is supported by numerous studies on knockout mice for ApoE and other animal models of atherosclerosis [63]. In these models, inhibition of endothelial NO production accelerates the formation of lesions in the aorta and the coronary arteries, and treatment with l-arginine preserves vessel morphology. Superoxide dismutase (SOD), catalase, glutathione peroxidase (GPx), and peroxiredoxins (Prxs) are expressed to counter cellular ROS [64]. Arteries experiencing disturbed blood flow and low shear stress at the curves and branches are susceptible to atherosclerotic lesions [5]. These regions contain a sensitive glycocalyx layer with an irregular alignment. Consequently, the low production of NOS and antioxidant enzymes compromise endothelium integrity. In exposure to certain predisposing risk factors such as hypertension, hyperglycemia, diabetes, ageing, hypercholesterolemia, and the mechanical stimuli owing to low shear stress, endothelium undergoes certain modifications which are characteristics for atherosclerotic initiation process [9, 65]. Morphological modification of endothelial cells and increased permeability to LDL particles thus allows penetration (transcytosis) and accumulation of ApoB, chylomicrons, and remnants of very-low-density lipoproteins (VLDLs) in the subendothelial space where they experience oxidative modifications by cytokines (monocyte chemotactic protein-1 (MCP-1)) [48]. The antithrombotic factors are compromised along with elevated vasoconstrictor and prothrombotic products via cell surface adhesion molecules such as intercellular adhesion molecule 1 (ICAM-1) and vascular cell adhesion molecule 1 (VCAM-1), hence elevating risk for inflammatory actions owing to Ox-LDL [66, 67]. These changes increase the adhesion of monocytes and penetration through the vascular wall which is modulated by cytokines and is augmented by interferon gamma and tumour necrosis factor-alpha [68, 69]. Studies report Ox-LDL as a key into the above-mentioned mechanism. In fact, Ox-LDL is chief in activating endothelium to secrete MCPs recruiting monocytes and T cells into endothelium.

NO plays a crucial part in protecting endothelium with its vasodilator property. It is also seen to reduce monocyte adhesion to the endothelium. Ox-LDL-induced reduction in the NO derived from the endothelium NOS is suggested as one of the causes of endothelial phenotypic changes [70]. Most of its activity is proposed to lectin-like oxidized LDL receptor 1 (LOX 1). LOX 1 is an Ox-LDL receptor found on endothelial cells and is likely to be overexpressed in atherosclerotic conditions.

There have been debates among chronology of events that lead to plaque formation. Since few scientists consider subendothelial retention of ApoB lipoproteins as the initiating factor in atherosclerosis, contradictory to which some authors suggest that everything starts with endothelial dysfunction [53]. High LDL level also marks its strong part in the argument. A plethora of research suggests that all these phenomena are more or less equally indispensable factors in the progression of atherosclerosis [71].

### 2.3. LDL Retention in the Subendothelium Elicits an Immune Response: Progression Stage

The entry of LDL particles in the subendothelium (tunica intima) followed by their retention through ApoB 100 binding to proteoglycans of the extracellular matrix is a key initiating factor in early atherogenesis. Oxidative modifications of LDL induce expression of cell adhesion molecules on the endothelial cells recruiting mainly monocytes and T lymphocytes into the inflamed arterial wall. Differentiation of monocytes into macrophage expresses scavenger receptors (CD36, SR-AI/II, and SR-BI) and LOX 1 (lectin-like Ox-LDL receptor 1) on the surface to recognize Ox-LDL [58, 59]. Various studies have established that to be recognized by a scavenger or oxidized receptors, the native LDL must be converted to Ox-LDL as discussed in previous section (due to its high affinity to scavenger receptors, SR) [72]. As soon as macrophages engulf massive Ox-LDL particles, foam cells are generated and a number of proinflammatory events take place: lipid retention, more oxidation of native LDL, release of proinflammatory cytokines, ROS, metalloproteases, and monocyte and Ox-LDL recruitment. Cytokines released by T lymphocytes and foam cells promote inflammation and ROS generation. Unsurprisingly, Ox-LDL along with these cells releases growth factors promoting SMC migration (via platelet drive growth factor and basic fibroblast growth factor) from tunica media into site region and abnormal proliferation (via insulin-like growth factor 1 and epidermal growth factor) that involves secretion of extracellular matrix proteins [73]. It is also seen that Ox-LDL drives SMCs to produce collagen and elastin which forms a necrotic core around the plaque increasing the lesion size at some point. It is important to mention that a lot of these events are more or less LOX 1 mediated [74]. Atherosclerotic plaque is a large necrotic core of foam cells, SMCs, collagen, calcium, and a thin fibrous cap preventing plaque from the bloodstream [75]. Hence, Ox-LDL plays a significant role in these events and moreover it is believed to induce apoptosis or at some point necrosis, favouring cellular debris deposits in the lesion area. Moreover, the ROS present in the plaque tends to induce cell death making the plaque unstable and rupture, which is the worst part of the progression.

### 2.4. Rupturing of Plaque: Thrombosis

A vulnerable plaque is subject to rupture. ROS degrades the fibrous wall of plaque via the release of matrix metalloproteinases (MMPs) [76] causing thrombus formation. Recent studies suggest a major difference in the lipid profiles of stable and unstable plaque considering former to be more susceptible to oxidation (due to increased PUFAs) and enhanced oxidation in the latter due to increased 18 : 0 containing lysophosphatidylcholine [77]. Rupturing of this plaque causes sudden expansion of the lesions leading to thrombus formation accounting for myocardial infarction, stroke, or sudden death [63, 64]. Looking more into the mechanism; Ox-LDL induces CD36 and P-selectin expression in the platelets activating them to further express LOX 1 for their adhesion on endothelium. Chemokines released from activated platelets mediate endothelium dysfunction, foam cell formation, and ROS production favouring progression of the plaque. Ox-LDL again makes the plaque scenario complex by platelet activation and adhesion on the endothelium which in turn blocks the NO production, which is said to be dysfunctional, yet again the cycle goes on [78].

## 3. Therapeutics to Block or Retard the Process: Endothelium Dysfunction to Thrombosis

**Figure 7:**
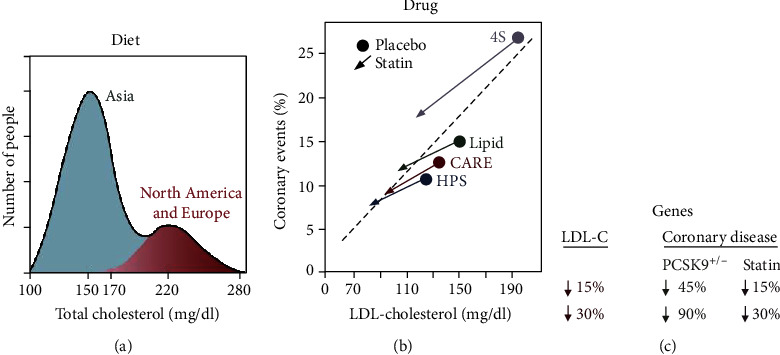
Diagram illustrating the effects of diet, drugs, and genes on plasma LDL and coronary disease. The figure is reproduced from Goldstein and Brown [35] (under the Creative Commons Attribution License/public domain) “(Reprinted (A Century of Cholesterol and Coronaries: From Plaques to Genes to Statins) with permission from Elsevier (License number 4825791117921)).” (a) Diet. Idealized depiction of the frequency distribution of plasma cholesterol levels in the human species as extrapolated from surveys of middle-aged people in major populations of the world. The higher the cholesterol level, higher the risk for coronary disease, as denoted by graded red shading. (b) Drugs. Frequency of coronary events plotted against plasma level of LDL cholesterol in four double-blind, placebo-controlled trials in which middle-aged people at risk for heart attacks were treated for 5 years with a statin or placebo. The number of subjects in each study was as follows: 4S Study, 4,444; LIPID, 9,014; CARE, 4,159; and HPS, 20,536. (c) Genes. Difference in risk for coronary disease in middle-aged people depending on whether plasma LDL cholesterol level is reduced over a lifetime (heterozygous loss of function of PCSK9) or for only 5 years (statin therapy).

The level and persistence of disease is the deciding factor for its treatment measures [79]. Some patients only require a lifestyle amendment and some have to undergo surgical procedures. HDL, antioxidant enzymes, diet, statins, and lipid lowering are foreseen as potent factors in preventing atherosclerosis. An illustration of effects of such parameters on CVDs is shown in Figure 7 [35]. Pharmacological interventions like angiotensin converting enzyme (ACE) inhibitors, statin insulin sensitizers and L-arginine, folates, and tetrahydrobiopterin are suggested to improve damaged endothelium which would resist LDL entry into subendothelium.

### 3.1. Lipid Management Is the Primary Prevention

**Figure 8:**
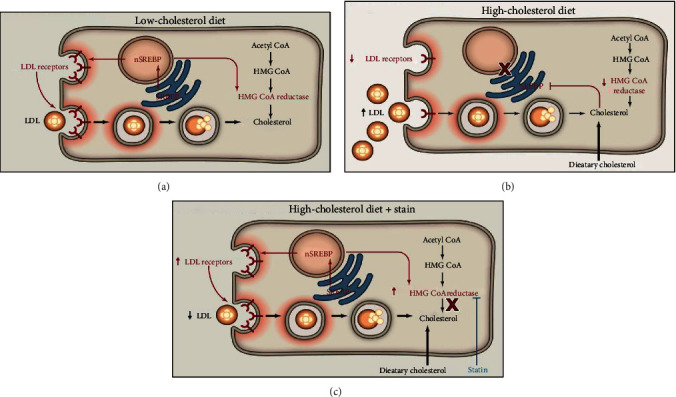
Hepatic responses to diet and statins mediated by the SREBP pathway. The figure is reproduced from Goldstein and Brown [35] [under the Creative Commons Attribution License/public domain] “(Reprinted (A Century of Cholesterol and Coronaries: From Plaques to Genes to Statins) with permission from Elsevier (License number 4825791117921)).” (a) Low-cholesterol diet. Proteolytic cleavage of SREBP is increased. The cleaved SREBP enters the nucleus to activate genes controlling cholesterol synthesis (including HMG CoA reductase) and uptake (LDL receptor). nSREBP, nuclear portion of cleaved SREBP. (b) High-cholesterol diet. Proteolytic cleavage of SREBPs is decreased, resulting in decreased nuclear SREBP and decreased activation of target genes. The decrease in LDL receptors produces an increase in plasma LDL. (c) High-cholesterol diet plus statin therapy. Statins inhibit HMG CoA reductase, causing a transient decrease in ER cholesterol. In response, SREBP cleavage is increased, and the resulting nuclear SREBP activates the genes for HMG CoA reductase and LDL receptor. The increased HMG CoA reductase is inhibited by the statin, and the increased LDL receptors lower plasma LDL.

As reviewed by Michos et al. [80] very recently, lipid management remains the primary prevention to atherosclerosis. If we look back into this review and perhaps in literature, the story always begins with high LDL cholesterol (LDL-C) which makes it the hallmark and a potent treatment target. Additionally, the CVD prevention guidelines published by the American College of Cardiology-America Heart Association recommends cholesterol management as a primary prevention by opting a healthy lifestyle [81]. Keeping in view the sedentary lifestyle and family history of CVDs as important risk factors, improvement in lifestyle and diet has been seen to reduce CVDs by about 50%. A healthy lifestyle here involves maintaining an ideal weight, keeping check on the blood sugar levels, regular exercise, and looking after calories intake. The notion “lower is better” goes exactly with the diet intake for a healthy lifestyle. Though merely management of lipids cannot completely prevent the disease, it does lower the risk to an extent. According to US Cholesterol Clinical practice guidelines (2018), statins should be the primary choice of pharmacological treatments for LDL-C considering the latter being critical in the therapeutics [82]. Statins tend to reduce about 50% LDL cholesterol via inhibiting 3-hydroxy-3-methylglutaryl-CoA reductase (HMG CoA), a rate limiting inhibitor of LDL-C. In atherosclerosis, there is an aberrant increase in the expression of MHC-II and thus, it is also considered as a chronic inflammatory disease. A study suggested that statins could function as immune suppressors by repressing the MHC-II expression and thereby, reducing the MHC-II-dependent T-lymphocyte activation and thus, statins could modulate the adaptive immunity [83]. Statins are seen to block the cholesterol synthesis by the activation of SREBP-2, a mechanism illustrated by Goldstein and Brown given in Figure 8 [35]. Compelling results from meta-analysis trials have revealed a lowering of about 22% risk of cardiovascular events with the reduction of a significant amount of LDL level [84]. Additionally, cardiac events like myocardial infarction and stroke can be prevented by lipid lowering with the use of high-intensity statins [85, 86].

### 3.2. HDL Retards the Atherosclerotic Process

**Figure 9:**
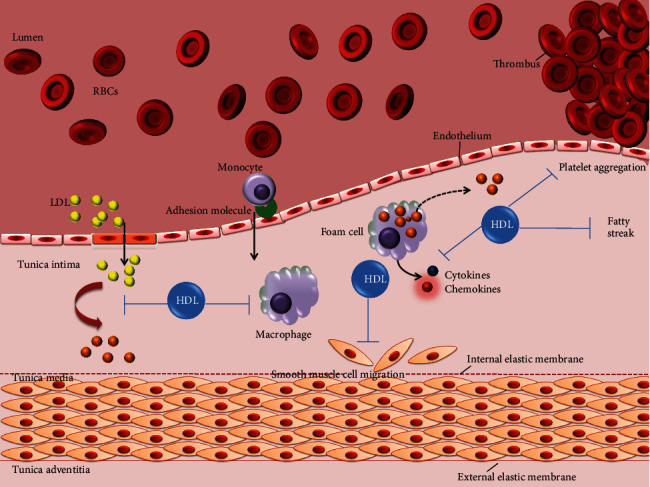
Schematic representation of HDL countering major atherosclerotic stages. HDL counters significant stages of atherosclerosis. First, HDL retards the oxidation process of LDL and helps in the efflux of cholesterol form macrophages, preventing it from becoming foam cells. Cytokines and growth factors released by T lymphocytes and foam cells induce SMC migration and proliferation that is again countered by HDL. Additionally, platelet migration and aggregation is prevented to the site of plaque. Hence, high HDL is antiatherosclerotic and comes into play as soon as the LDL penetrates the dysfunctional endothelium.

HDL along with its major lipid poor apolipoprotein A-I (ApoA-I) aids in removing cholesterol from foam cells for clearance by the liver. The efflux from macrophage foam cells is the first step towards reverse cholesterol transport that lowers further inflammation preventing atherosclerosis burden [73, 74]. A line of epidemiological trails has revealed that HDL accounts for douching lipid oxidation via antioxidant enzymes like Lp-PLA2, LCAT, and PON1 along with ApoA-I which helps in removing hydroperoxides from cells and LDL [87]. HDL signalling via endothelial cell receptors like SR-BI helps in enhancing NO production, thus maintaining endothelium homeostasis [88]. Additionally, it prompts cholesterol efflux from platelets preventing platelet aggregation and eventually thrombosis [89]. Accumulation and retention of Ox-LDL is the key initiating factor in chemotaxis and monocytes invasion. This LDL modification primarily by endothelium, SMCs, and macrophages in the lesion area would suggest that cells actively modify LDL. LDL is either oxidized nonenzymatically by transition metal ions, hemin, and many other catalysts or by enzymes within the artery wall. HDL obviates the oxidative modification of LDL employing proteins circulating on HDL itself. The most important protein is paraoxonase 1 (PON1) that confers an ability to prevent LDL oxidation by enhancing the antioxidative function of HDL [87]. Additionally, ApoA-I aids in reducing lipid hydroperoxides to lipid hydroxides owing to oxidation in its methionine residues [90]. LCAT and lipoprotein-associated phospholipase A2 (Lp-PLA2) have also been reported to douche oxidative mechanisms as suggested in mice experiments [91]. Nevertheless, HDL could absorb oxidizing lipids to prevent the further process of LDL oxidative modification.  Figure 9 gives illustrations of key events where HDL comes in to play. Hence, antioxidative capacity conferred by HDL proteins ensures protective measures to retard the atherosclerotic lesion and more extensive research is required to better understand it to use it as a therapeutic target.

### 3.3. Role of Dietary Antioxidants in Treating Atherosclerosis

Antioxidants scavenge free radicals (reactive oxygen/nitrogen species) and reduce the probability of oxidative stress [92]. Antioxidants can be synthesized by a cell itself or can be taken exogenously. Antioxidants synthesized by a cell include glutathione, uric acid, caeruloplasmin, ferritin, transferrin, or lactoferrin, whereas vitamin E, vitamin C, flavonoids, and carotenoids come from the diet [93–95]. The endogenous nonenzymatic antioxidants like GSH, uric acid, bilirubin, coenzyme Q, and lipoic acid can be present intracellularly or extracellularly and serve as the primary defence system against imbalanced redox stress [96, 97]. Glutathione, a cofactor for glutathione peroxidase (GPx) scavenges hydroxide, hypochlorous acid, and peroxinitrite, thus modulates size of the atherosclerotic lesion [98]. Coenzyme Q improves endothelial function by scavenging peroxyl radicals [99]. Bilirubin, uric acid, and lipoic acid have shown remarkable scavenging properties towards oxidants, hence improving endothelium and decreasing inflammatory actions [100–102].

The secondary defence mechanism involves exogeneous antioxidants like vitamin C, vitamin E, vitamin A, vitamin B, carotenoids, or polyphenols [100, 103–105]. As mentioned above, the native LDL, when turned into Ox-LDL becomes atherogenic and this oxidative modification in LDL is due to imbalanced ROS. Vitamin E, a lipid soluble antioxidant, aids in neutralizing free radicals hence retarding LDL oxidation. Moreover, vitamin E inhibits the expression of CD36, SR-BI, and protein kinase C to reverse foam cell formation and SMC proliferation along with further associated complications of the CVD [106, 107]. It is also observed that vitamin E modulates the expression of connective tissue growth factors and adhesion molecules (VCAM 1, ICAM 1) on endothelial cells and helps in the prevention endothelium dysfunction and internalization of LDL molecules into the intima. In contrast, some clinical trials have given completely opposite outcomes where studies showed almost no effect of vitamin E doses (50 mg-300 mg/day) in the CVD patients [103, 106]. No decrease of stroke and myocardial infarction was observed in population with CVDs. While the outcomes of the trails remain contradictory, more studies are anticipated to build a strong conclusion. Vitamin C (ascorbate) helps to stabilize cell membranes by scavenging peroxyl radicals, ROS, and superoxide radicals. It enhances NO bioavailability, thus improving endothelium and vasodilation. In vitro experiments have shown lowered Ox-LDL generation on treating with ascorbate (vitamin C), while depletion of the same is related to atherosclerosis in vivo [100]. Ascorbate has also been seen to reduce endothelium dysfunction which again is a key initiation factor of the CVD [94]. But several other clinical trials have shown contradictory results where no significant relationship between vitamin C and the reversal of atherosclerosis was found. A study group of 29,133 men (50-69 yrs) was given synthetic *α*-tocopherol (50 mg/day) and *β*-carotene (20 mg/day) for ~6 years, and no effect was seen in improving heart condition. In fact, the risk of lung carcinoma and ischemic heart disease was increased [108]. Similar study on people without any CVD was carried out for 4 years in which incidences of angina pectoris slightly increased as an adverse effect of the supplementation [109]. In another study on group of smokers and nonsmokers (45-74 yrs), even including the vitamin A in a combination with above two had no effect. In an additional study, *β*-carotene (50 mg/day) could not show any positive effect on patients supplemented for 4-8 years (27-84 yrs) [110]. Combination of vitamin E (30 mg/day), vitamin C (120 mg/day), *β*-carotene (6 mg/day), selenium (100 *μ*g/day), and zinc (20 mg/day) was given to 13,017 middle-aged people for 7.5 years, and it was found that combined supplementation had very slight effect on men in improving cardiovascular health and no effect at all was seen on women participants [111]. Although dietary supplements have some role in preventing atherosclerosis in mice and human trails [112], yet a very little is known to conclude exactly how much, when, and what should be included in the diet to have the best effect.

### 3.4. Antioxidant Enzymes Are Critical in Atherosclerosis Prevention

In addition to the above-mentioned antioxidants, the cells also express antioxidant enzymes like catalase, superoxide dismutase (SOD), thioredoxin reductase (TrxR), peroxiredoxins (Prxs), glutathione peroxidase (GPx), glutathione reductase, and glutathione-S-transferase which maintain redox homeostasis [113–115]. The most crucial enzymes in such mechanisms include peroxiredoxins (Prxs) and glutathione peroxidase 4 (Gpx4). Peroxiredoxins (Prxs; 1-6) are proteins that have cysteine residues that reduce peroxides, in turn, getting oxidized into sulfenic acid [116, 117]. Prx1 and Prx2 exacerbate atherosclerosis on deletion in ApoE mice [118]. While Prx4 inhibits the disease from being overexpressed in ApoE mice, Prx6 has no such effect [119]. Glutathione peroxidases convert glutathione to glutathione disulfide and reduce lipid peroxides to water [120]. The most crucial role is played by Gpx4. Loss of GPx4 activity in the cellular membranes leads to ferroptosis, which is a regulated nonapoptotic cell death process and it is enhanced by the accumulation of ROS in a lipid-rich environment [121]. As mentioned above, PUFAs with labile bis-allylic hydrogen atoms are prone to lipid peroxidation and it is also the hallmark of ferroptosis [122]. According to Yang et al., there are two key drivers in ferroptosis induced by PUFA peroxidation: lipoxygenase and phosphorylase kinase G2 (PHKG2). The study suggested that PUFA peroxidation is caused by lipoxygenases via PHKG2 and consequently the inhibition of catalytic selenocysteine in GPx4 causes the accumulation of the hydroperoxides leading to cell death [123]. Ferroptosis is linked to several diseases like cancer and atherosclerosis and needs clear mechanistic insights for therapeutic interventions [124, 125].

## 4. Outlook

Endothelium under oxidative stress primes to the formation of oxidized cholesterol (oxysterols) as a result of low-density lipoprotein (cholesterol carrier through the blood) oxidation. The oxidation is driven by oxygen, nitrogen, or other reactive species which brings up the oxidative or nitrosative stress in uncontrolled measures. Certain irritants like toxins from smoking (nicotine and carbon monoxide), high blood pressure due to hypertension, high level of low-density lipoproteins, HDL dysfunction, obesity, and diabetes mellitus alter the endothelium lining causing permeation and accumulation of LDL particles into the subendothelial space. Retention of LDL particles brings about a lot of factors into play which upsurges the risk for fatty streak development and further complication into atherosclerosis progression. What come to the rescue are monocytes but turns out to be exaggerating the process. Hence, the immune system more likely induces inflammation making things more complicated. Antioxidant enzymes and genes, however, are potent measures to counter ROS to prevent lipid peroxidation. Since dietary antioxidants and statins have also shown effective activity in vivo, these can be effectively prescribed for treatment and cholesterol lowering. Additionally, maintaining a healthy lifestyle is a key to homeostasis and a healthy cardiovascular system. Although, an enormous amount of research has been conducted on the above events, yet a clear understanding of therapeutic measures is required.

## Figures and Tables

**Table 1:** Risk factors to atherosclerosis.

Hyperlipidemia
Homocysteine levels in plasma
Hypertension
Smoking
Low or impaired HDL
Family history of CVDs
Obesity
Aging
Male sex
Physical inactivity
Stress/depression
High cholesterol
Sedentary lifestyle
Unhealthy diet
Psychological and socioeconomic factors
